# Identification of a novel variant in *MLH1* intron causing aberrant splicing associated with Lynch syndrome

**DOI:** 10.1093/gastro/goaf104

**Published:** 2025-12-09

**Authors:** Ai-Xin Liu, Kai-Hua Liu, Rong-Yun Guo, Jian-Ming Ying, Shuang-Mei Zou, Lin Dong

**Affiliations:** Department of Pathology, National Cancer Center/National Clinical Research Center for Cancer/Cancer Hospital, Chinese Academy of Medical Sciences and Peking Union Medical College, Beijing, P. R. China; Geneseeq Research Institute, Nanjing Geneseeq Technology Inc., Nanjing, Jiangsu, P. R. China; Geneseeq Research Institute, Nanjing Geneseeq Technology Inc., Nanjing, Jiangsu, P. R. China; Department of Pathology, National Cancer Center/National Clinical Research Center for Cancer/Cancer Hospital, Chinese Academy of Medical Sciences and Peking Union Medical College, Beijing, P. R. China; Department of Pathology, National Cancer Center/National Clinical Research Center for Cancer/Cancer Hospital, Chinese Academy of Medical Sciences and Peking Union Medical College, Beijing, P. R. China; Department of Pathology, National Cancer Center/National Clinical Research Center for Cancer/Cancer Hospital, Chinese Academy of Medical Sciences and Peking Union Medical College, Beijing, P. R. China

## Introduction

Lynch syndrome (LS) is one of the most common hereditary cancer syndromes, increasing risk for colorectal, endometrial, ovarian and other cancers. It is an autosomal dominant genetic disease caused by pathogenic germline variants in mismatch repair (MMR) genes, including *MLH1*, *MSH2*, *MSH6*, and *PMS2*, or deletion in the *EPCAM* gene [1]. Pathogenic variants in these genes impair the correction of nucleotide mismatches caused by replication slippage errors, resulting in characteristic microsatellite instability (MSI) in tumors. While next-generation sequencing has uncovered a large number of variants, many are classified as variants of uncertain significance (VUS), complicating clinical interpretation [2]. Without robust evidence confirming the pathogenicity of the VUS, it is difficult to make a definitive diagnosis or offer appropriate genetic counselling for at-risk carriers.

Aberrant mRNA splicing contributes to approximately 10%–15% of all genetic diseases [3]. Variants located within specific regions of MMR genes can disrupt normal splicing, leading to the production of abnormal transcripts and functionally defective proteins that drive tumorigenesis. Notably, splice-site variants at non-canonical positions frequently evade definitive pathogenic classification without functional validation at the RNA level [4]. For this purpose, RNA sequencing has emerged as a powerful diagnostic tool for detecting splicing defects, providing a robust platform for interpretation of such variants [5].

In this study, we report a novel *MLH1* splice-site variant (c.790 + 5G>C) identified in a Chinese family affected by LS. Splicing analysis, based on DNA and RNA sequencing, was performed to evaluate the pathogenicity of this variant, generating molecular evidence critical for clinical diagnosis and genetic counselling for at-risk family members.

## Case report

**Figure 1.**
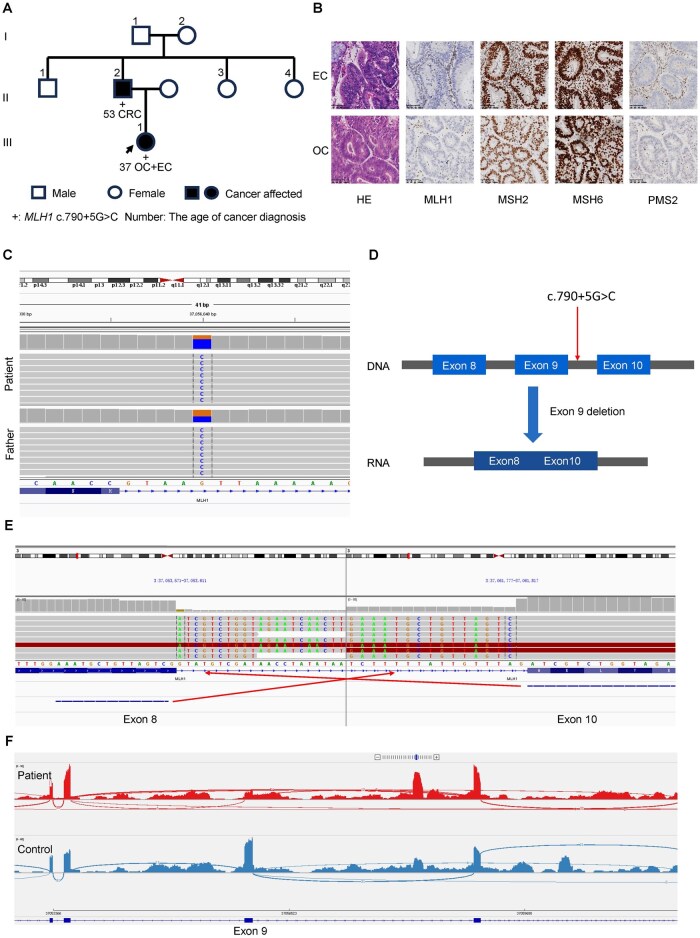
Clinical and molecular features of the pedigree. (A) Family pedigree. The proband is indicated by an arrow. The number represents the age at cancer diagnosis. The proband (III-1) was diagnosed with ovarian cancer (OC) and endometrial cancer (EC) at the age of 37 years. Her father (II-2) was diagnosed with colorectal cancer (CRC) at the age of 53 years. (B) MMR protein expression of the proband. Loss of MLH1 and PMS2 proteins was detected. All images are captured at 200× magnification. (C) Germline testing results. DNA next-generation sequencing (NGS) of the proband (III-1) and her father (II-2) reveals a germline variant in *MLH1* (c.790 + 5G>C). (D) Schematic depiction of *MLH1* c.790 + 5G>C variant and its consequence on the RNA level, revealing exon 9 skipping. (E) IntegrativeGenomeViewer snapshot of exon 9 skipping. (F) Sashimi plots of *MLH1* splicing patterns in the normal pattern (control) and patient by RNA-sequencing. The patient has very few junction reads to exon 9 compared with the normal pattern (control).

The proband was a 37-year-old woman presenting with a 7.3-cm left ovarian mass and an endometrial polyp. She was treated with a left adnexectomy and endometrial biopsy. Postoperative histopathological examination confirmed endometrioid adenocarcinoma of the left ovary and endometrium. The proband received four cycles of adjuvant chemotherapy, followed by a reoperation of hysterectomy. Her father had a history of colorectal cancer diagnosed at 53 years old (Figure 1A). No other malignancies were reported in the family.

The proband’s clinical presentation and family history fulfilled the revised Bethesda guidelines. The PREMM5 model yielded an overall predicted probability of 21.0% for carrying a germline *MMR* mutation, exceeding the 2.5% threshold and justifying further evaluation for LS. Immunohistochemical (IHC) analysis demonstrated loss of nuclear staining in MLH1 and PMS2 proteins, with intact expression of MSH2 and MSH6 proteins (Figure 1B). MSI test confirmed an MSI-high status in the tumor tissue.

A novel splice-site variant of *MLH1* (*MLH1* [NM_000249.3]: c.790 + 5G>C) was identified by using DNA sequencing. This germline variant was detected in both the proband and her father, but was absent in the general population according to the Exome Aggregation Consortium (ExAC) database (Figure 1C). Reference to the International Society for Gastrointestinal Hereditary Tumors (InSiGHT) DNA variant database revealed no record of this specific variant; however, two similar variants at the same position—c.790 + 5G>A and c.790 + 5G>T—have been reported as pathogenic. *In silico* prediction indicated that the c.790 + 5G>C variant was likely to disrupt RNA splicing. To confirm this, RNA sequencing was performed by using the proband’s normal tissue, and a fusion transcript of *MLH1* exons 8–10 was observed, confirming exon 9 skipping at the RNA level (Figure 1D–F). This aberrant splicing event caused a frameshift during translation, introducing a premature stop codon at position 238, which was likely a target for nonsense-mediated mRNA decay. Functional analysis revealed that the resulting truncated peptide of 237 amino acids harbored a complete loss of the C-terminus functional domain. Based on the cumulative evidence, the variant was classified as pathogenic according to the ACMG/AMP guidelines, fulfilling criteria PVS1, PS1, PM2, and PP4.

The proband was diagnosed with Lynch syndrome–associated cancers. She continued with chemotherapy after surgeries, and no metastasis was detected during 6 months of follow-up. Her father, who underwent radical resection, remains disease-free to date.

## Discussion

Variants of *MLH1* account for approximately 43% of all reported variants associated with LS, a substantial proportion of which are classified as VUS [6]. Determining the pathogenicity of these VUS is critically important for establishing an accurate diagnosis and guiding appropriate genetic counselling and clinical surveillance for carriers.

A subset of *MLH1* VUS, particularly those located at the 5′ splice sites, is suspected to affect RNA splicing [5]. The +1 and +2 positions within introns are canonical splice sites that play an indispensable role in splicing recognition; consequently, variants at these positions are typically classified as pathogenic. In contrast, single-nucleotide substitutions at the +5 position are more challenging to interpret. Although frequently reported as VUS, their impact on splicing is variable, complicating clinical assessment [7].

In current genetic counselling practice, patients with a relevant family history and MMR protein deficiency identified by IHC are recommended for further evaluation for LS. While concurrent loss of MLH1 and PMS2 protein expression typically triggers reflex testing for *MLH1* promoter hypermethylation and *BRAF* V600E mutations to exclude sporadic cases, this case highlights that cryptic splicing variants represent an underrecognized confounder in methylation-negative cohorts [8]. Although conventional DNA sequencing is effective for detecting sequence-level variants, it has limited sensitivity in identifying functionally significant splicing variants. *In silico* prediction tools offer preliminary insights into potential splicing disruptions; however, owing to the complexity of splicing regulation, experimental validation remains indispensable [4, 9]. RNA-based assays, including RNA sequencing and minigene splicing assays, serve as essential tools. In our case, concurrent MLH1/PMS2 IHC loss and a VUS in MLH1 initially created diagnostic uncertainty, which was resolved only through RNA-level analysis. This underscores a critical gap in current practice: 23% of MLH1-deficient, methylation-negative cases harbor non-canonical splice variants, requiring specialized RNA-based testing for accurate classification [10]. Therefore, RNA-level assays should be strongly considered for evaluating VUS affecting splice sites as a supplement to conventional LS screening strategies.

In conclusion, we identified a novel intronic *MLH1* variant (c.790 + 5G>C) in one Chinese family and functionally validated its pathogenicity through RNA splicing analysis. This finding expands the spectrum of variants in *MLH1* and provides valuable evidence for informing genetic counselling and diagnostic strategies for LS-associated cancers.

## References

[1] Ballester V , Cruz-Correa M. How and when to consider genetic testing for colon cancer? Gastroenterology 2018;155:955–9. 30148981 10.1053/j.gastro.2018.08.031

[2] Horton C , Hoang L, Zimmermann H et al Diagnostic outcomes of concurrent DNA and RNA sequencing in individuals undergoing hereditary cancer testing. JAMA Oncol 2024;10:212–9. 37924330 10.1001/jamaoncol.2023.5586 PMC10625669

[3] Pagenstecher C , Wehner M, Friedl W et al Aberrant splicing in MLH1 and MSH2 due to exonic and intronic variants. Hum Genet 2006;119:9–22. 16341550 10.1007/s00439-005-0107-8

[4] Takahashi M , Furukawa Y, Shimodaira H et al Aberrant splicing caused by a MLH1 splice donor site mutation found in a young Japanese patient with lynch syndrome. Fam Cancer 2012;11:559–64. 22766992 10.1007/s10689-012-9547-1

[5] Brandão RD , Mensaert K, López-Perolio I et al; KConFaB Investigators. Targeted RNA-seq successfully identifies normal and pathogenic splicing events in breast/ovarian cancer susceptibility and lynch syndrome genes. Int J Cancer 2019;145:401–14. 30623411 10.1002/ijc.32114 PMC6635756

[6] Tournier I , Vezain M, Martins A et al A large fraction of unclassified variants of the mismatch repair genes *MLH1* and *MSH2* is associated with splicing defects. Hum Mutat 2008;29:1412–24. 18561205 10.1002/humu.20796

[7] Buratti E , Chivers M, Královičová J et al Aberrant 5′ splice sites in human disease genes: mutation pattern, nucleotide structure and comparison of computational tools that predict their utilization. Nucleic Acids Res 2007;35:4250–63. 17576681 10.1093/nar/gkm402 PMC1934990

[8] Soukarieh O , Gaildrat P, Hamieh M et al Exonic splicing mutations are more prevalent than currently estimated and can be predicted by using in silico tools. PLoS Genet 2016;12:e1005756. 26761715 10.1371/journal.pgen.1005756 PMC4711968

[9] Li J , Ni H, Wang X et al Association of a novel frameshift variant and a known deleterious variant in MMR genes with lynch syndrome in Chinese families. World J Surg Oncol 2024;22:36. 38280988 10.1186/s12957-024-03309-5 PMC10821544

[10] Bouras A , Legrand C, Kourda J et al From variant of unknown significance to likely pathogenic: characterization and pathogenicity determination of a large genomic deletion in the MLH1 gene. Mol Genet Genomic Med 2023;11:e2231. 37350751 10.1002/mgg3.2231 PMC10496038

